# Response of leaf and fine roots proteomes of *Salix viminalis* L. to growth on Cr-rich tannery waste

**DOI:** 10.1007/s11356-016-7026-1

**Published:** 2016-06-09

**Authors:** Agata Zemleduch-Barylska, Gabriela Lorenc-Plucińska

**Affiliations:** Institute of Dendrology Polish Academy of Sciences, Parkowa 5, 62-035 Kórnik, Poland

**Keywords:** *Salix viminalis*, Solid tannery waste, Chromium, Phytoremediation, Proteomic analysis, Leaves, Roots

## Abstract

The tannery industry is a major source of anthropogenic chromium (Cr) contamination due to the large amounts of solid waste produced and its problematic management. The unique composition of tannery waste, usually high concentrations of Cr and other metals as well as organic matter and nutrients, makes it a great risk for soil and water environment but also a possible effective fertilizer for non-food plants that can tolerate metals. The goal of this study was to understand the adaptation mechanism of *Salix viminalis* to growth on Cr-rich tannery waste from an active landfill. We used a proteomic approach to identify leaf and fine roots proteins altered by tannery waste as compared to control soil conditions. We found no obvious symptoms of oxidative stress in leaves or fine roots. Proteomic results indicated some changes in metabolism, with increases in energy production processes and their greater efficiency for leaves rather than root development. Comparison between *S. viminalis* and *P.* × *canescens* response to tannery waste suggested that *S. viminalis* is not suitable for remediation of Cr-contaminated areas of a tannery waste landfill site.

**Electronic supplementary material:**

The online version of this article (doi:10.1007/s11356-016-7026-1) contains supplementary material, which is available to authorized users.

## Introduction

Despite effort searching for cleaner and cheaper treatment technologies, landfill and land application are still the most common ways to dispose of tannery sludge and tanned solid waste (Lόpez-Luna et al. 2009; Silva et al. 2013; Pati et al. 2014; Ali et al. 2015). The resulting dumping sites pose a risk for soil and groundwater due to leachate of chromium (Cr), other metals, and chemicals (Dixit et al. 2015). However, tannery sludge is widely used as a fertilizer in some countries, as it introduces additional nutrients to the soil and may improve some soil properties (Nakatani et al. 2012; Silva et al. 2013; Chand et al. 2015). The influence of tannery waste on the growth and physiology of plants is ambiguous due to complex characteristics of this substrate. Concentration of Cr and its redox state are among the main determinants of the waste toxicity to plants (Gill et al. 2015; Patel and Patra 2015).

The effects of specified Cr(III) and Cr(VI) salt doses on the growth, metabolic status, and oxidative stress responses of different plants have been frequently studied (Labra et al. 2006; Yu and Gu 2007; Bah et al. 2010; Sharmin et al. 2012; Wang et al. 2013). There are some similarities between the effects of applying Cr salts and tannery waste on plant physiology, for example, the generation of free radicals and lipid peroxidation in leaves and roots and activation or inhibition of their antioxidative defense system (Sinha et al. 2007a, b; Chandra et al. 2009; Gill et al. 2015; Patel and Patra 2015). Despite parallels, the effects of Cr-rich tannery waste on plant growth and physiology cannot be explained by only the presence of Cr ions—they strongly depend on Cr mobility and bioavailability, as well as on other organic/inorganic compounds present in waste (Lόpez-Luna et al. 2009). Waste toxicity can be mitigated (or even completely suppressed) by high levels of organic matter, a suitable pH or high concentrations of nutrients (Giachetti and Sebastiani 2007; Lόpez-Luna et al. 2009; Silva et al. 2013), the consequences of which seem to be increased growth and productivity for some plants (Giachetti and Sebastiani 2006; Sinha et al. 2007a, b; Gupta and Sinha 2009; Shukla et al. 2011). Grey poplar (*Populus* × *canescens* Sm.) was able to grow on solid tannery waste from an active landfill site without a decline in sapling biomass or elevated oxidative stress, despite high accumulation of Cr (Zemleduch and Lorenc-Plucińska 2011). Moreover, proteomic data suggested enhanced nitrogen (N) uptake and metabolism and possible changes in cell wall composition, which may have been key features allowing it to grow on such waste (Zemleduch-Barylska and Lorenc-Plucińska 2015).

*Populus* spp. and *Salix* spp. are both potential candidates for phytoremediation of tannery-affected sites (Tognetti et al. 2004; Giachetti and Sebastiani 2007; Quaggiotti et al. 2007; Shukla et al. 2011). Our previous studies showed that growth of grey poplar and willow (*Salix viminalis* L.) planted on tannery waste was not severely affected (*p* > 0.05) (Zemleduch and Lorenc-Plucińska 2011)—only the dry mass of willow roots decreased compared to plants grown on control soil. Both tree species contained higher concentrations of N, sulfur (S), calcium (Ca), sodium (Na), iron (Fe), and Cr in the roots than control plants, while phosphorus (P) and zinc (Zn) concentrations were lower. At the same time, *S. viminalis* accumulated ca. 2.7 times less Cr in the roots than *P.* × *canescens* (276 and 759 mg kg^−1^ dry mass, respectively) and seemed incapable of translocating it to the leaves (Zemleduch and Lorenc-Plucińska 2011). Although poor translocation of Cr from roots to shoots has been previously observed in *Salix* spp. (Pulford et al. 2001), the other differences in reaction to growing on tannery waste between *P.* × *canescens* and *S. viminalis* were puzzling. This was especially so considering that many studies have shown greater metal tolerance and better remediation capacity [e.g., for cadmium (Cd), copper (Cu), Zn, and lead (Pb)] of willow compared to poplar (Fischerová et al. 2006; Bissonnette et al. 2010; Zacchini et al. 2009).

Therefore, the aim of the present study was to understand the molecular and physiological mechanisms involved in response of *S. viminalis* to Cr-rich tannery waste. We focused on a proteomic approach to identify essential proteins, in which their abundance in the leaves and roots of willow grown on tannery waste from an active landfill was affected. In addition, malondialdehyde, superoxide dismutase, catalase, and enzymes involved in ascorbate-glutathione cycle and low-molecular-weight antioxidants in plant were simultaneously analyzed. The activity of antioxidative system is presumed as the main mechanism of adaptation to tannery waste (Sinha et al. 2007b; Gupta and Sinha 2009; Patel and Patra 2014). *S. viminalis* was grown on the same tannery waste and at the same time as *P. × canescens*. Hence, the second objective of our work was to assess the likely difference in the response mechanism of *S. viminalis* to Cr-waste to the one that was previously reported in *P.* × *canescens* (Zemleduch-Barylska and Lorenc-Plucińska 2015). We hope that insights into the molecular and physiological mechanisms involved in the Cr stress response of these two species explain their different effectiveness in tolerating and accumulating Cr. Overall, our study aimed to clarify the potential use of *S. viminalis* and *P.* × *canescens* for the remediation of Cr-contaminated tannery waste landfill.

## Materials and methods

### Plant material and growth experiment

*S. viminalis* L. was planted on solid tannery waste originated from an active landfill site or unpolluted soil (control) taken from the Institute of Dendrology, Polish Academy of Sciences (ID PAS) poplar plantation. Details about the landfill site, the waste, and the soil characteristics were described in Zemleduch and Lorenc-Plucińska (2011). The tannery waste comprised pressed sediment obtained from tannery effluent treated with appropriate coagulants, such as Pix 113 [Fe(III) sulfate], Pax 15 (polyaluminum chloride) and lime, and mixed with chrome trimmings, fleshings, and shavings as well as unfinished leather splits. Cr(III) was present at phytotoxic levels: 23 026 mg kg^−1^ dry weight (DW) of total and 156 mg kg^−1^ DW of bioavailable forms of Cr. Concentrations (mg kg^−1^ DW) of nutrients and heavy metals in control soil and tannery waste were total N (900 and 18 100, respectively), S (2000 and 39 300), P (374 and 5418), K (1577 and 455), magnesium (Mg, 840 and 3583), Ca (1269 and 126 801), Cu (4.97 and 27.17), Fe (6242 and 17 165), Zn (23 and 173), nickel (Ni, 2 and 25), Pb (14 and 45), and organic matter (15 100 and 407 000), as well as some physical parameters, such as pH (5.21 and 7.34), conductivity (mS cm^−1^) (21.6 and 2480), cation exchange capacity (CEC, in cmol kg^−1^) (1.69 and 67.76), and clay content (%) (2 and 6). Bioavailable forms of all elements were also higher in the waste than in control soil.

Dormant hardwood cuttings (20–25 cm long) of *S. viminalis* (collected from 1-year-old stems on stool beds established at cutting orchards, ID PAS) were grown for 16 weeks in a shaded poly-tunnel, in 2.5-L pots filled with soil or tannery waste. Saplings were hand-watered daily or when required using tap water. At the end of the experiment, fine roots (diameter < 2 mm) and healthy leaves were sampled from at least six plants per growth variant, mixed, frozen in liquid nitrogen, and stored at −80 °C for biochemical and molecular analyses. Mycorrhiza associated with roots, were regarded as a part of the roots.

### Lipid peroxidation

To estimate intensity of lipid peroxidation in leaves and fine roots, malondialdehyde (MDA) content was measured using thiobarbituric acid (TBA) reaction (Heath and Packer 1968). MDA concentration (C) was calculated using the formula C (μmol L^−1^) = 6.45(A_532_ − A_600_) − 0.56A_450_ (Yang et al. 2009).

### Antioxidant analyses

Antioxidative enzyme extracts from leaves and fine roots were prepared as described in Zemleduch-Barylska and Lorenc-Plucińska (2015). Protein content was determined according to Bradford (1976). Superoxide dismutase (SOD) was measured according to McCord and Fridovich (1969), catalase (CAT) according to Aebi (1984), ascorbate peroxidase (APX) according to Nakano and Asada (1981), and guaiacol peroxidase (GPOD) by the method of Zimmerlin et al. (1994). Glutathione reductase (GR) was determined according to the methodology of Edwards et al. (1994) and dehydroascorbate reductase (DHAR) and monodehydroascorbate reductase (MDAR) activities according to Krivosheeva et al. (1996). Reduced (GSH) and oxidized glutathione (GSSG) contents were determined according to Griffith (1980). Determinations of SOD, APX, GPOD, DHAR, and MDAR in fine roots were not performed because of their poor growth on tannery waste and thus a lack of sufficient replicates.

### Two-dimensional gel electrophoresis

For each organ (leaves and fine roots) and treatment (soil and tannery waste), at least three independent protein extractions and two-dimensional gel electrophoresis (2-DE) analyses were performed. Protein extracts were prepared with two-step procedure that combined trichloroacetic acid (TCA)/acetone precipitation and phenol extraction, as described in Zemleduch-Barylska and Lorenc-Plucińska (2015). The 2-DE gels staining with Coomassie Brilliant Blue R-250, image scanning, and their analysis in Image Master 2D Platinum Software 6.0 (GE Healthcare, Uppsala, Sweden) were carried out according to standard protocols described in detail in Zemleduch-Barylska and Lorenc-Plucińska (2015).

Protein spots with at least a twofold variation at *p* < 0.05, identified as differentially abundant proteins in both growth conditions (soil and tannery waste), were excised manually from the gels and subjected to mass spectrometry (MS) analysis.

### Mass spectrometry and protein identification

Proteins in excised spots were trypsin digested and analyzed by liquid chromatography coupled to a LQT FT ICR mass spectrometer (Hybrid-2D-Linear Quadrupole Ion Trap Fourier Transform Ion Cyclotron Resonance Mass Spectrometer, Thermo Electron Corp, San Jose, CA) in the Mass Spectrometry Laboratory of the Institute of Biochemistry and Biophysics, PAS (Warsaw, Poland). Procedure and MS data processing were as described in Zemleduch-Barylska and Lorenc-Plucińska (2015). The functions of the unknown or predicted proteins were predicted according to protein BLAST search (http://blast.ncbi.nlm.nih.gov/Blast.cgi). The biological significance of the identified proteins was assessed based on ontological data from the Kyoto Encyclopedia of Genes and Genomes (KEGG) database (http://www.genome.jp/kegg/) and from UniProt (http://www.uniprot.org/).

### Statistical analysis

Results were subjected to Fisher’s least significant difference (LSD) test, using STATISTICA 10 software (StatSoft Inc., USA), to compare mean values (*n* = 6) from treated and control samples. Differences were considered significant at *p* ≤ 0.05.

## Results and discussion

### Oxidative stress symptoms in leaves and fine roots

**Fig. 1:**
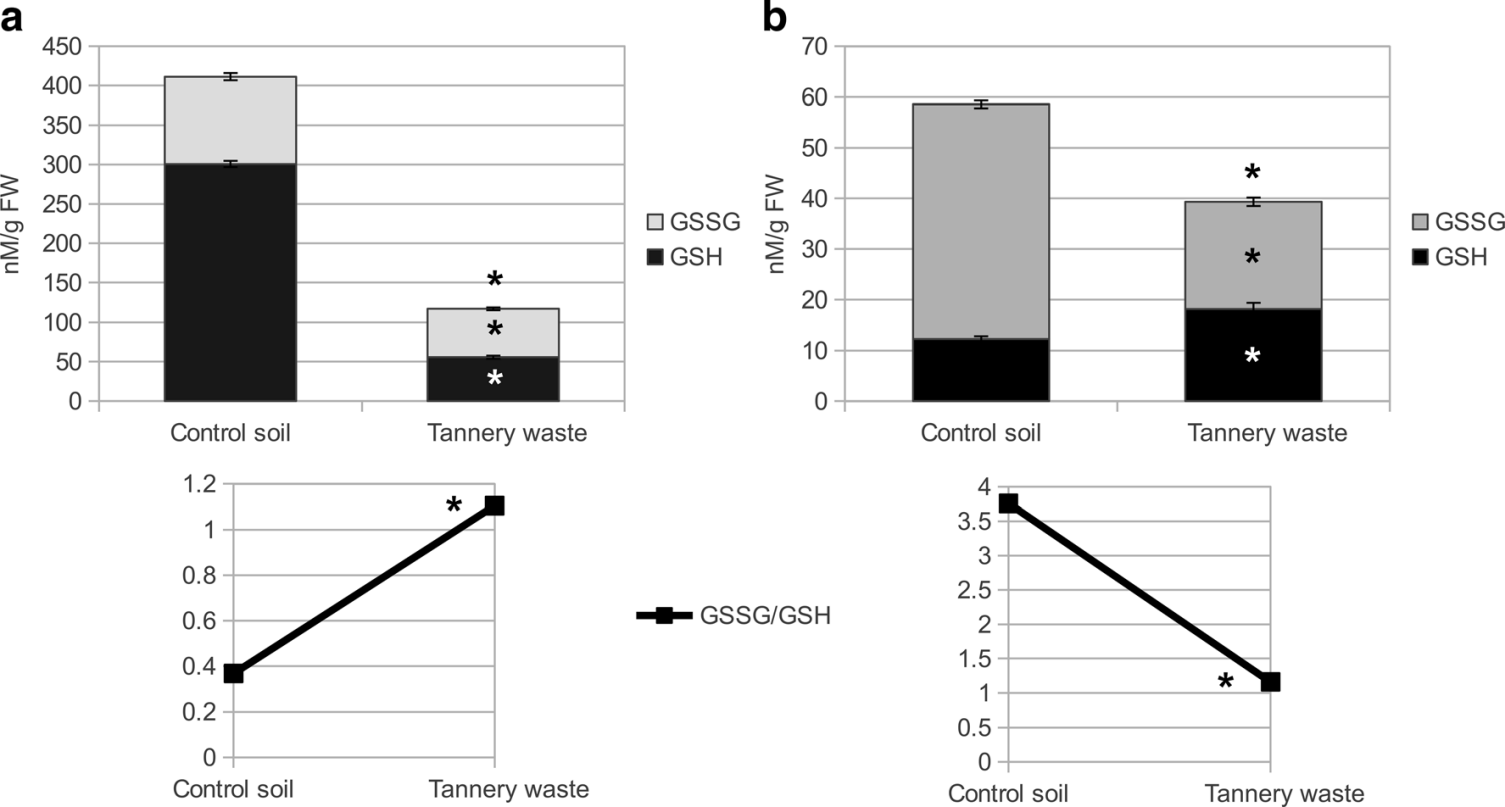
Concentration of reduced (GSH) and oxidized glutathione (GSSG) (nM g^−1^ FW) and GSSG/GSH ratio in leaves (**a**) and roots (**b**) of *S. viminalis* planted on control soil and on tannery waste. Results represent means ± standard error. Values marked with *asterisk* are significantly different from controls (*p* > 0.05)

Oxidative stress is a secondary stress accompanying adverse environmental conditions including excess of metals in the soil (Gill and Tuteja 2010; Bhaduri and Fulekar 2012). The level of MDA is often regarded as an indicator of oxidative stress and lipid peroxidation, disrupting the function and integrity of biological membranes (Gupta and Sinha 2009; Gill and Tuteja 2010; Bhaduri and Fulekar 2012). No significant changes were found in the content of MDA in either leaves or fine roots of *S. viminalis* grown on tannery waste (Table 1). The activity of catalase, which catalyzes the decomposition of hydrogen peroxide (Gill et al. 2015), was even lower than in control plants (Table 1). However, some other common antioxidative enzymes—e.g., APX, GPOD, DHAR, and MDHAR (Gill and Tuteja 2010)—had increased activity in the leaves but not SOD (Table 1). These results may indicate effective response to oxidative stress in the willow grown on tannery waste. GR, which regenerates GSH from GSSG (Jozefczak et al. 2012), was decreased in both leaves and fine roots (Table 1). This result corresponded to glutathione content in leaves, where growth on tannery waste caused a decrease in its total and reduced form concentrations and increased the ratio of oxidized to reduced forms (GSSG/GSH) (Fig. 1a). In the roots, the lower GR activity seemed to be somehow balanced because total glutathione concentration decrease was due to decreases in the oxidized form only, and GSSG/GSH ratio was also lower than in controls (Fig. 1b). Cr ions can react with the GSH sulfhydryl group to form an unstable complex, thus contributing to the deposition of Cr in roots and reducing its translocation to stems and leaves (Zeng et al. 2012).

**Table 1:** Malondialdehyde (MDA) concentration (μmol g^−1^ FW) and antioxidative enzymes—superoxide dismutase (SOD), catalase (CAT), ascorbate peroxidase (APX), guaiacol peroxidase (GPOD), dehydroascorbate reductase (DHAR), monodehydroascorbate reductase (MDHAR), and glutathione reductase (GR) activity (for SOD U mg^−1^ protein, for the rest nkat mg^−1^ protein)—in leaves and fine roots of *S. viminalis* planted on control soil and fresh tannery waste. Results represent means ± standard error; *p* values—significance of differences between control soil and tannery waste; *ns* indicates *p* > 0.05

Organ	Variant	MDA	SOD	CAT	APX	GPOD	DHAR	MDAR	GR
Leaves	Control soil	0.049 ± 0.004	749 ± 16	51.17 ± 1.5	3.5 ± 0.17	18.18 ± 0.15	0.35 ± 0.03	0.235 ± 0.008	0.317 ± 0.017
	Tannery waste	0.054 ± 0.001	479 ± 18	16.83 ± 1.17	4.83 ± 0.17	26.25 ± 0.1	1.083 ± 0.033	0.28 ± 0.006	0.143 ± 0.012
	p	ns	<0.00001	<0.00001	0.003	<0.00001	<0.00001	0.001	0.0045
Fine roots	Control soil	0.018 ± 0.001		3750 ± 50					4 ± 0.17
	Tannery waste	0.0198 ± 0.001		1315 ± 12					2.55 ± 0.13
	p	ns		<0.00001					0.001

The results of the analysis of oxidative stress in leaves and roots of *S. viminalis* were in contrast to those for *P.* × *canescens* in the same conditions, where there was no change in MDA level in leaves but a decrease in fine roots, together with increased CAT activity in both tissues. In poplar grown on tannery waste, there was an increase in glutathione concentration in both leaves and fine roots and also an opposite reaction to willow for the GSSG/GSH ratio, accompanying decreased GR activity (Zemleduch-Barylska and Lorenc-Plucińska 2015). Observed differences between those species could be the result of different metabolic approaches.

### Changes in proteomes of leaves and fine roots

**Fig. 2:**
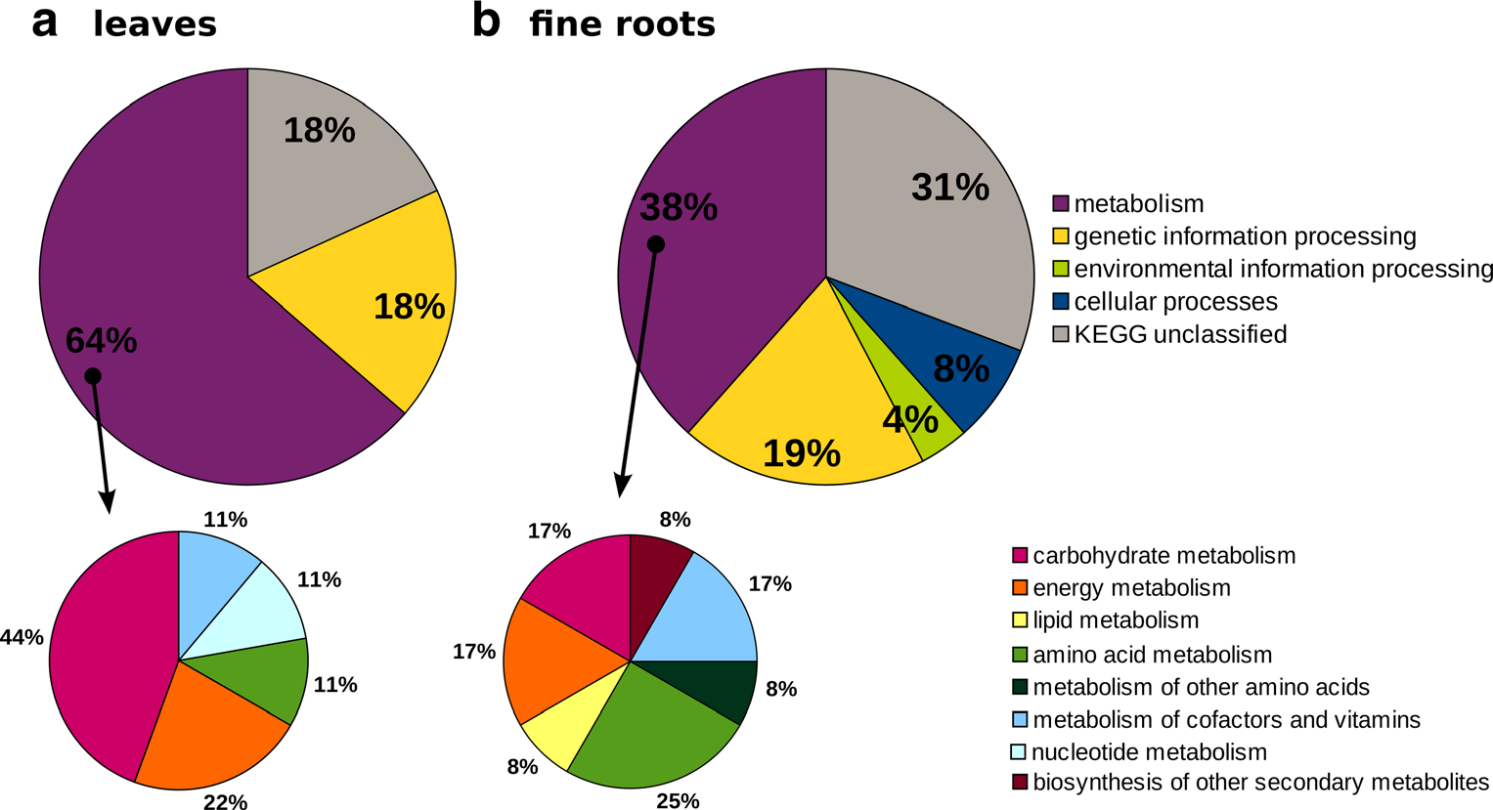
Functional classification of proteins identified in the leaves (**a**) and fine roots (**b**) of *S. viminalis*

Quantitative analysis revealed 11 protein spots with significant differences (twofold change, *p* < 0.05) in abundance between control and fresh tannery waste grown willow plants for leaves and 30 spots for fine roots. These numbers roughly corresponded to 1.4 % of leaf and 5.5 % of fine root proteomes. For *P. × canescens* in parallel conditions (considering twofold change, *p* < 0.05), this was only 1 and 1.5 % of leaf and root proteomes, respectively (Zemleduch-Barylska and Lorenc-Plucińska 2015). Tables 2 and 3 include the complete, annotated lists of *S. viminalis* proteins identified with MS (10/11 spots from leaves and 24/30 spots from roots), and Fig. 2 presents their functional classification.

**Table 2:** Proteins identified by MS/MS analysis in the *leaves* of *Salix viminalis* grown on control soil or on tannery waste. Change ↑ or ↓ indicates overabundant or less abundant proteins, respectively, after the growth on tannery waste (*p* < 0.05). tw means a protein spot was present in tannery waste growth variant only. Fold = fold change (ratio). % prot seq cover = % of protein sequence covered by the matched peptides. Main KEGG class abbreviations: M metabolism, GIP genetic information processing. Main GO class abbreviations: BP biological process, MF molecular function, CC cellular component

Spot nb	Change^a^	Fold^a^	Accession nb^b^	Name^b^	Score^b^	Nb of peptides mached^b^	% prot seq cover^b^	pI exp/theor^a,b^	Mass [kDa] exp/theor^a,b^	BLAST^c^	KEGG classification^d^	GO classification^e^
**27**	↓	2.26	gi|224095228	Predicted protein [*Populus trichocarpa*]	845	14	31	6.59/6.59	51.1/50	cysteine desulfurase/transaminase [*Populus trichocarpa*]	M: *metabolism of cofactors and vitamins; thiamine metabolism GIP: *folding, sorting and degradation; sulfur relay system	BP: *[2Fe-2S] cluster assembly MF: *ATP binding and cysteine desulfurase activity and pyridoxal phosphate binding and zinc ion binding
**5**	tw	–	gi|224058573	Predicted protein [*Populus trichocarpa*]	255	4	21	5.15/5.64	27.1/25.04	Osmotin precursor, putative [*Ricinus communis*]	–	BP: *defense response to bacterium and incompatible interaction and defense response to fungus and response to other organism and response to salt stress CC: *endomembrane system
**9**	tw	–	gi|15220397	Lactoylglutathione lyase, putative/glyoxalase I, putative [*Arabidopsis thaliana*]	422	9	18	5.7/6.97	33.76/39.43		M: *carbohydrate metabolism; pyruvate metabolism	BP: *response to cold MF: *lactoylglutathione lyase activity and metal ion binding CC: * chloroplast; chloroplast stroma; stromule; thylakoid; thylakoid lumen
**12**	↑	2.82	gi|224072248	Predicted protein [*Populus trichocarpa*]	1987	37	41	5.19/5.1	83.18/73.7	Luminal binding protein [*Gossypium hirsutum*]	GIP: *folding, sorting, and degradation; protein export and protein processing in endoplasmic reticulum	MF: *ATP binding
**13**	tw	–	gi|224146286	Predicted protein [*Populus trichocarpa*]	287	5	10	5.2/4.8	21.67/18.4	Nuclear transport factor 2 (NTF2) family protein [*Arabidopsis thaliana*]	–	BP: *protein import into nucleus and transport
**18**	↑	3.21	gi|118489670	Unknown [*Populus trichocarpa* x *Populus deltoides*]	3471	27	66	5.52/5.4	30.17/24	Inorganic pyrophosphatase [*Populus trichocarpa*]	M: *energy metabolism; oxidative phosphorylation	BP: *phosphate-containing compound metabolic process MF: *inorganic diphosphatase activity and magnesium ion binding CC: *cytoplasm
**20**	↑	2.00	gi|224109060	Predicted protein [*Populus trichocarpa*]	1085	12	32	5.87/8.25	45.47/50.3	Phosphoglycerate kinase [*Populus trichocarpa*]	M: *carbohydrate metabolism; glycolysis/gluconeogenesis *Energy metabolism; carbon fixation in photosynthetic organisms	BP: *glycolytic process MF: *ATP binding and phosphoglycerate kinase activity
**22**	↑	2.11	gi|224060560	Predicted protein [*Populus trichocarpa*]	1747	33	35	5.93/6.7	64.14/63	d-3-Phosphoglycerate dehydrogenase, putative [*Ricinus communis*]	M: *amino acid metabolism; Glycine, serine, and threonine metabolism	BP: *L-serine biosynthetic process MF: *NAD binding and phosphoglycerate dehydrogenase activity
**24**	↑	2.00	gi|12585330	RecName: Full = Phosphoglucomutase, cytoplasmic; Short = PGM; AltName: Full = Glucose phosphomutase	932	12	25	5.62/5.49	73.4/63.37		M: *carbohydrate metabolism; glycolysis/gluconeogenesis and pentose phosphate pathway and galactose metabolism and starch and sucrose metabolism and amino sugar and nucleotide sugar metabolism *Nucleotide Metabolism; purine metabolism	BP: *glucose metabolic process MF: *magnesium ion binding and phosphoglucomutase activity CC: *cytoplasm
**33**	↑	2.76	gi|224113035	Predicted protein [*Populus trichocarpa*]	661	17	27	5.78/5.84	63.74/56.7	Phosphoglucosamine mutase family protein [*Populus trichocarpa*]	M: carbohydrate metabolism; amino sugar and nucleotide sugar metabolism	–

^a^Data obtained during comparison of protein profiles in IMP 6.0

^b^Data obtained during identification of proteins in the Mascot database (http://www.matrixscience.com)

^c^Identification of unknown or predicted proteins according to the NCBI BlastP database (http://blast.ncbi.nlm.nih.gov/Blast.cgi)

^d^Classification from the Kyoto Encyclopedia of Genes and Genomes (KEGG) database (http://www.genome.jp/kegg/)

^e^Gene ontology (GO) classification from the UniProt database (http://www.uniprot.org/ )

**Table 3:** Proteins identified by MS/MS analysis in the *fine roots* of *Salix viminalis* grown on a control soil or on tannery waste. Change ↑ or ↓ indicates overabundant or less abundant proteins, respectively, after the growth on tannery waste (*p* < 0.05). tw or cs means a protein spot was present in tannery waste or control soil, respectively, growth variant only. Fold = fold change (ratio). % prot seq cover = % of protein sequence covered by the matched peptides. Main KEGG class abbreviations: M metabolism, GIP genetic information processing, CP cellular processes, EIP environmental information processing. Main GO class abbreviations: BP biological process, MF molecular function, CC cellular component

Spot nb	Change^a^	Fold^a^	Accesion nb^b^	Name^b^	Score^b^	Nb of peptides mached^b^	% prot seq cover^b^	pI exp / theor^a,b^	mass [kDa] exp / theor^a,b^	BLAST^c^	KEGG Classification^d^	GO Classification^e^
**2**	cs	-	gi|118484162	unknown [Populus trichocarpa]	1373	24	39 %	5.6 / 8.50	29.73 / 27.70	Probable ATP synthase 24 kDa subunit, mitochondrial [Arabidopsis thaliana]	**M;** *Energy Metabolism; Oxidative phosphorylation	-
**3**	cs	-	gi|224114988	predicted protein [Populus trichocarpa]	200	3	14 %	5.8 / 5.88	29.91 / 28.30	stem-specific protein tsjt1, putative [Jatropha curcas]	-	**BP**; *glutamine metabolic process **CC**; *cytosol
**5**	cs	-	gi|225449497	PREDICTED: similar to HSC70-1 (heat shock cognate 70 kDa protein 1); ATP binding isoform 1 [Vitis vinifera]	236	5	12 %	6.37 / 5.17	64.1 / 71.5		**GIP;** *Transcription; Spliceosome *Folding, Sorting and Degradation; Protein processing in endoplasmic reticulum **CP**; * Transport and Catabolism; Endocytosis	**MF**; *ATP binding
**6**	↓	2.10	gi|224053010	predicted protein [Populus trichocarpa]	2186	23	35 %	5.7 / 9.20	43.73 / 41.6	enoyl-[acyl-carrier-protein] reductase [Populus trichocarpa]	**M;** *Lipid Metabolism; Fatty acid biosynthesis	-
**11**	cs	-	gi|224093330	predicted protein [Populus trichocarpa]	122	3	14 %	4.75 / 4.91	21.57 / 20.6	Kunitz-type protease inhibitor KPI-F9 [Populus trichocarpa x Populus deltoides]	-	**MF;** *endopeptidase & inhibitor activity **CC**; *apoplast, cell wall
**15**	↓	2.28	gi|118484484	unknown [Populus trichocarpa]	1308	3	18 %	5.32 / 5.79	20.27 / 17.5	type II peroxiredoxin [Populus trichocarpa]	-	**MF**; *antioxidant activity & oxidoreductase activity
**16**	cs	-	gi|118487795	unknown [Populus trichocarpa]	269	8	20 %	5.76 / 6.3	41.9 / 35.9	annexin, putative [Ricinus communis]	-	**MF**; *peroxidase activity & protein homodimerization activity & calcium-dependent phospholipid binding & calcium ion binding
**17**	cs	-	gi|2501578	Probable pyridoxal biosynthesis protein PDX1; AltName: Ethylene-inducible protein HEVER	182	4	16 %	6.29 / 6.26	40.79 / 33.39		**M;** *Metabolism of Cofactors and Vitamins; Vitamin B6 metabolism	**BP**; *pyridoxal phosphate biosynthetic process; vitamin B6 biosynthetic process **CC**; *pyridoxal 5'-phosphate synthase (glutamine hydrolysing) activity
**24**	cs	-	gi|224131686	vitamin-b12 independent methionine synthase, 5-methyltetrahydropteroyltriglutamate-homocysteine [Populus trichocarpa]	666	16	20 %	6.59 / 6.26	95.9 / 84.9		**M;** *Amino Acid Metabolism; Cysteine and methionine metabolism	-
**25**	cs	-	gi|224131618	predicted protein [Populus trichocarpa]	420	3	12 %	5.17 / 5.24	28.81 / 26.25	carboxymethylenebutenolidase, putative [Ricinus communis]	-	**MF**; *hydrolase activity & transferase activity & transferring acyl groups other than amino-acyl groups
**26**	cs	-	gi|224101413	predicted protein [Populus trichocarpa]	1226	8	45 %	5.57 / 5.60	20.84 / 17.60	eIF5A1 [Populus deltoides]	**GIP;** *Translation; Translation factors	**BP**; *positive regulation of translational elongation & positive regulation of translational termination & translational frameshifting **CC**; *ribosome binding & translation elongation factor activity & translation initiation factor activity
**32**	↓	2.28	gi|224076645	predicted protein [Populus trichocarpa]	1524	7	25 %	5.81 / 6.08	25.54 / 21.6	Minor allergen Alt a, putative [Ricinus communis] ~ Flavoprotein wrbA, putative [Ricinus communis] ~ quinone reductase-like [Vitis vinifera]	-	**BP**; *negative regulation of transcription
**38**	↓	2.25	gi|224093760	predicted protein [Populus trichocarpa]	404	6	18 %	6.5 / 6.34	38.88 / 36.3	Anx1 [Gossypium hirsutum]	-	**MF;** *kinase activity & protein kinase activity & protein serine/threonine kinase activity & calcium-dependent phospholipid binding & calcium ion binding
**40**	↓	3.03	gi|224118628	predicted protein [Populus trichocarpa]	560	8	32 %	5.87 / 5.37	26.76 / 25.8	proteasome subunit beta type 6,9, putative [Ricinus communis]	**GIP;** *Folding, Sorting and Degradation; Proteasome	**BP**; *proteolysis involved in cellular protein catabolic process **MF**; *threonine-type endopeptidase activity **CC**; *cytoplasm & nucleus & proteasome core complex
**41**	↓	3.42	gi|283135906	DHAR class glutathione transferase DHAR2 [Populus trichocarpa]	1952	15	45 %	5.7 / 5.50	27.12 / 23.6		**M;** *Metabolism of Other Amino Acids; Glutathione metabolism	**MF**; *transferase activity
**44**	cs	-	gi|224140239	predicted protein [Populus trichocarpa]	658	10	20 %	5.46 / 5.46	62.04 / 51.84	DNA helicase, putative [Ricinus communis]	**GIP;** *Replication and Repair; Non-homologous end-joining	**MF**; *ATP binding & ATP-dependent 5'-3' DNA helicase activity **CC**; *nucleus
**46**	↓	2.45	gi|118487795	unknown [Populus trichocarpa]	1475	11	37 %	6 / 6.3	42.09 / 35.9	annexin, putative [Ricinus communis]	-	**MF;** *peroxidase activity & protein homodimerization activity & calcium-dependent phospholipid binding & calcium ion binding
**63**	cs	-	gi|224134068	predicted protein [Populus trichocarpa]	329	8	13 %	6 / 6.43	55.68 / 51.73	glutamate-1-semialdehyde 2,1-aminomutase [Populus trichocarpa]	**M;** *Metabolism of Cofactors and Vitamins; Porphyrin and chlorophyll metabolism	-
**70**	cs	-	gi|224061286	predicted protein [Populus trichocarpa]	211	6	33 %	5.24 / 5.48	27.47 / 24.28	AtRABA1f (Arabidopsis Rab GTPase homolog A1f); GTP binding [Arabidopsis thaliana]	**EIP;** *Signaling Molecules and Interaction; GTP-binding proteins	**BP**; *protein transport & small GTPase mediated signal transduction **MF**; *GTP binding **CC**; *intracellular
**10**	tw	-	gi|224111564	mitochondrial beta subunit of F1 ATP synthase [Populus trichocarpa]	433	11	23 %	5 / 5.9	68.9 / 59.9		**M;** *Energy Metabolism; Oxidative phosphorylation	-
**18**	↑	2.80	gi|74419004	glyceraldehyde-3-phosphate dehydrogenase [Populus maximowiczii x Populus nigra]	805	10	33 %	6.79 / 6.76	49.54 / 37.2		**M;** *Carbohydrate Metabolism; Glycolysis / Gluconeogenesis	**BP**; *glucose metabolic process **MF**; *NAD binding & NADP binding & oxidoreductase activity, acting on the aldehyde or oxo group of donors, NAD or NADP as acceptor
**33**	tw	-	gi|224120086	predicted protein [Populus trichocarpa]	542	13	21 %	4.6 / 5.2	87.58 / 75.4	heat shock protein, putative [Ricinus communis]	**GIP;** *Transcription; Spliceosome *Folding, Sorting and Degradation; Protein processing in endoplasmic reticulum **CP;** *Transport and Catabolism; Endocytosis	**BP**; *protein folding **MF**; *ATP binding
**49**	↑	2.09	gi|224139168	predicted protein [Populus trichocarpa]	542	11	17 %	5.9 / 5.5	78.69 / 65.9	pyruvate decarboxylase [Populus trichocarpa]	**M;** *Carbohydrate Metabolism; Glycolysis / Gluconeogenesis *Amino Acid Metabolism; Tryptophan metabolism	**MF**; *carboxy-lyase activity & magnesium ion binding & thiamine pyrophosphate binding
**53**	↑	3.46	gi|224082496	predicted protein [Populus trichocarpa]	246	4	14 %	5.49 / 5.39	61.43 / 39.28	peroxidase [Nicotiana tabacum]	**M;** *Amino Acid Metabolism; Phenylalanine metabolism *Biosynthesis of Other Secondary Metabolites; Phenylpropanoid biosynthesis	**BP**; *cellular oxidant detoxification & hydrogen peroxide catabolic process & plant-type cell wall organization & response to oxidative stress **MF**; *heme binding & metal ion binding; peroxidase activity **CC**; *extracellular region & plant-type cell wall

^a^Data obtained during comparison of protein profiles in IMP 6.0.

^b^Data obtained during identification of proteins in the Mascot database (http://www.matrixscience.com)

^c^Identification of unknown or predicted proteins according to the NCBI BlastP database (http://blast.ncbi.nlm.nih.gov/Blast.cgi)

^d^Classification from the Kyoto Encyclopedia of Genes and Genomes (KEGG) database (http://www.genome.jp/kegg/)

^e^Gene Ontology (GO) classification from the UniProt database (http://www.uniprot.org/ )

**Fig. 3:**
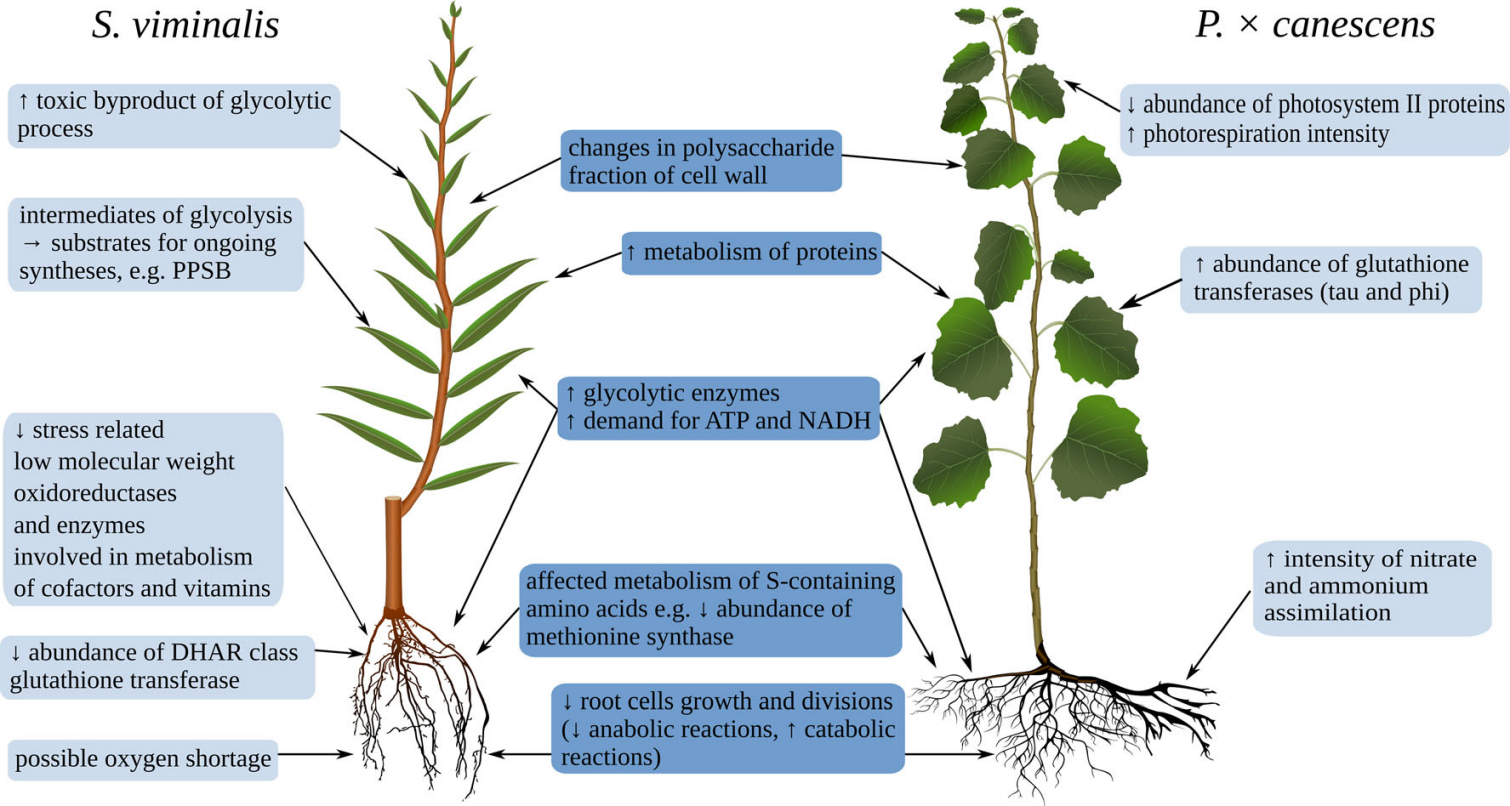
Comparison of effects on *S. viminalis* and *P*. × *canescens* growth on tannery waste. All conclusions were drawn from the results of functional classification of leaf and root proteins that were identified as variable between growth on control soil and on fresh tannery waste. Data for *P*. × *canescens* are from Zemleduch-Barylska and Lorenc-Plucińska (2015)

Both in leaves and fine roots, fresh tannery waste caused overabundance of enzymes related with glycolysis (see spots 20, 24, and 18 in Tables 2 and 3). The changes in the abundance of mitochondrial ATP synthase subunit in roots and inorganic pyrophosphatase in leaves (spots 2 and 18 in Tables 3 and 2) suggest the impact of waste on the oxidative phosphorylation process. This indicates increased demand for energy and reducing agents, commonly observed in stress conditions (Bah et al. 2010; Sharmin et al. 2012; Wang et al. 2013), as well as in tannery waste grown *P.* × *canescens* (Fig. 3). In leaves, we also observed greater abundance of lactoylglutathione lyase responsible for the degradation of methylglyoxal—a toxic by-product of the glycolytic process (spot 9 in Table 2) (Hossain et al. 2012). While, in roots, increased amount of pyruvate decarboxylase (spot 49 in Table 3) may indicate the redirection of pyruvate to a path leading to fermentation due to oxygen shortage (Shiao et al. 2002). However, intermediates of glycolysis could be used as substrates for ongoing syntheses, such as the phosphorylated pathway of serine biosynthesis (PPSB), an important pathway during environmental stress (Muñoz-Bertomeu et al. 2009). This hypothesis is in agreement with the observation in leaves of overabundance of d-3-phosphoglycerate dehydrogenase catalyzing one of the PPSB reactions (spot 22 in Table 2).

Fresh tannery waste affected the metabolism of S-containing amino acids in *S. viminalis* roots, an effect similar to that observed in *P.* × *canescens* (Fig. 3). Tannery waste contained more total S and sulfate-S than control soil (3.9 and 0.2 % and 2.7 and 0.1 %, respectively), and uptake of S ions by roots seemed not to be affected by Cr (S concentrations in waste or soil grown willow were 0.3 and 0.2 %, respectively). However, we found a reduction in amount of methionine synthase and glutathione transferase (spots 24 and 41 in Table 3), which use GSH to regenerate the reduced form of ascorbate (Jozefczak et al. 2012). Interestingly, these results were accompanied by decreased GSH concentration in roots of *S. viminalis* (Fig. 1b).

Our thesis about the lack of induction of oxidative stress in *S. viminalis* grown on tannery waste seemed to be confirmed also by depression of enzymes involved in metabolism of cofactors, vitamins, and stress-related low-molecular-weight proteins with oxidoreductase activity—for example, type II peroxiredoxin, and pyridoxal biosynthesis protein PDX1 (spots 15 and 17 in Table 3). Of note, pyridoxal 5ʹ-phosphate (vitamin B_6_) and its derivatives are among the most potent antioxidants (Leuendorf et al. 2010).

An example of *S. viminalis* adaptation to growth on fresh tannery waste could be an increased abundance of enzymes involved in the biosynthesis of cell wall components. In leaves, we found an overabundance of protein from phosphoglucosamine mutase family (spot 33 in Table 2), indicating changes in the wall polysaccharide fraction. We reached similar conclusions for *P.* × *canescens*, where there was an up-regulation of enzymes related to metabolism of UDP sugars, which are substrates for cell wall polysaccharide biosynthesis (Fig. 3). Such reactions in both tree species suggest changes in cell wall functioning that possibly lead to better protection from external factors or sequestration of xenobiotics within its structure. The second mechanism may work by Cr displacing Ca^2+^ and/or other cations from their binding sites in cell walls (Scoccianti et al. 2006).

The other symptoms of adaptation to tannery waste in leaf tissue were also visible in metabolism of proteins themselves. For factors involved in transport, sorting, and processing of proteins, an increase in relative protein abundance was found. These included luminal binding protein and nuclear transport factor 2 family protein (spots 12 and 13 in Table 2). Interestingly, according to Xu et al. (2013), endoplasmic reticulum (ER) luminal binding protein (BiP2) could have a role in alleviation of Cd^2+^-induced ER stress and programmed cell death in tobacco cells.

That trends of enhanced processing and movement of protein molecules in leaf cells, and the reduced biosynthesis or regeneration of damaged proteins and increased protein degradation in roots, were common to both *S. viminalis* and *P.* × *canescens* grown on tannery waste (Fig. 3).

In *S. viminalis* fine roots, there was a decrease in relative amounts of proteins involved in signal transduction into the cell and regulation of its growth, including Rab GTPase homolog A1f (spot 70 in Table 3) and annexins (spots 16 and 38) (Talukdar et al. 2009). Tannery waste also affected the abundance of proteins involved in genetic information processing, causing a decrease in eIF5A1 translation factor, DNA helicase, heat shock cognate 70 kDa protein 1, Kunitz-type protease inhibitor KPI-F9, and proteasome subunit beta type 6,9 (spots 26, 44, 5, 11, and 40 in Table 3). Similar effects on some proteins related to cell division, heat shock proteins (HSPs), or other chaperones as well as translation factors were also observed in other plants after Cr treatment (Bah et al. 2010; Sharmin et al. 2012). Such results seem to explain reduced growth and development of *S. viminalis* roots in these conditions and suggest that energy is used for adaptation and maintenance of metabolic processes, rather than for cell division and root growth.

## Conclusion

We found that fresh tannery waste affected the physiology of fine roots more than of leaves of *S. viminalis*. We observed increased energy demand as well as possible alterations in cell wall functioning and protein metabolism in leaves. This suggested deep rearrangement of metabolism in adapting the plant to increased concentrations of both Cr and nutrients in the waste. Effectiveness of this adaptation was illustrated by increased biomass of leaves and stems. There was a different effect in fine roots, where tannery waste caused a decreased abundance of proteins related to gene expression. Here, energy seemed to be invested in the maintenance of metabolic processes instead of cell division and development. The insights from proteomic analysis of *S. viminalis* and *P.* × *canescens*, both grown in analogous conditions, indicated some common reactions of trees to growth on Cr-rich tannery waste, such as changes in cell wall functioning and S metabolism. However, there were also some distinct reactions. Willow showed no indication of negative effects on photosynthesis, but there were symptoms of possible oxygen shortage in fine roots. Moreover, in comparison to *P.* × *canescens*, *S. viminalis* seemed incapable of efficient use of the N contained in tannery waste.

*Populus* spp. and *Salix* spp., both members of Salicaceae, have been proposed for phytoremediation of metal-contaminated sites (Tognetti et al. 2004; Giachetti and Sebastiani 2007; Ranieri and Gikas 2014). Higher survival rate and better remediation capacity of willow were shown by Bissonnette et al. (2010) and Fischerová et al. (2006). However, taking into account the similarities and differences between *S. viminalis* and *P.* × *canescens* in their ability for Cr accumulation and translocation, biomass production as well as changes at biochemical and molecular levels, we suggest that *S. viminalis* is not suitable for remediation of Cr-contaminated areas of a tannery waste landfill site. It should be noted that our results were from pot trials and so require field validation.

## Electronic supplementary material

Below is the link to the electronic supplementary material.

ESM 1 (PDF 13095 kb)
