# Supplementary material for: Response of leaf and fine roots proteomes of Salix viminalis L. to growth on Cr-rich tannery waste
Source: Environ Sci Pollut Res Int. 2016 Jun 9;23(18):18394–406. doi: 10.1007/s11356-016-7026-1 (PMC5026714; doi:10.1007/s11356-016-7026-1)

Supplementary material

Response of leaf and fine roots proteomes of *Salix viminalis* L. to growth on Cr-rich tannery waste

Environmental Science and Pollution Research

Agata Zemleduch-Barylska*, Gabriela Lorenc-Plucińska

Institute of Dendrology Polish Academy of Sciences, Parkowa 5, 62-035 Kórnik, Poland

*Corresponding author: Agata Zemleduch-Barylska

Tel.: +48 697418025; fax: +48 618170166

E–mail address: agata.zemleduch@wp.pl

Supplementary Fig. 1. Representative 2-DE gel obtained for the **leaves** of *S. viminalis* grown on a control soil or tannery waste. Differentially abundant protein spots (at least twofold variation at $p < 0.05$) identified with MS analysis are marked with arrows. Histograms show the distribution of relative spots volumes in each growth conditions (a = tannery waste, b = control soil).

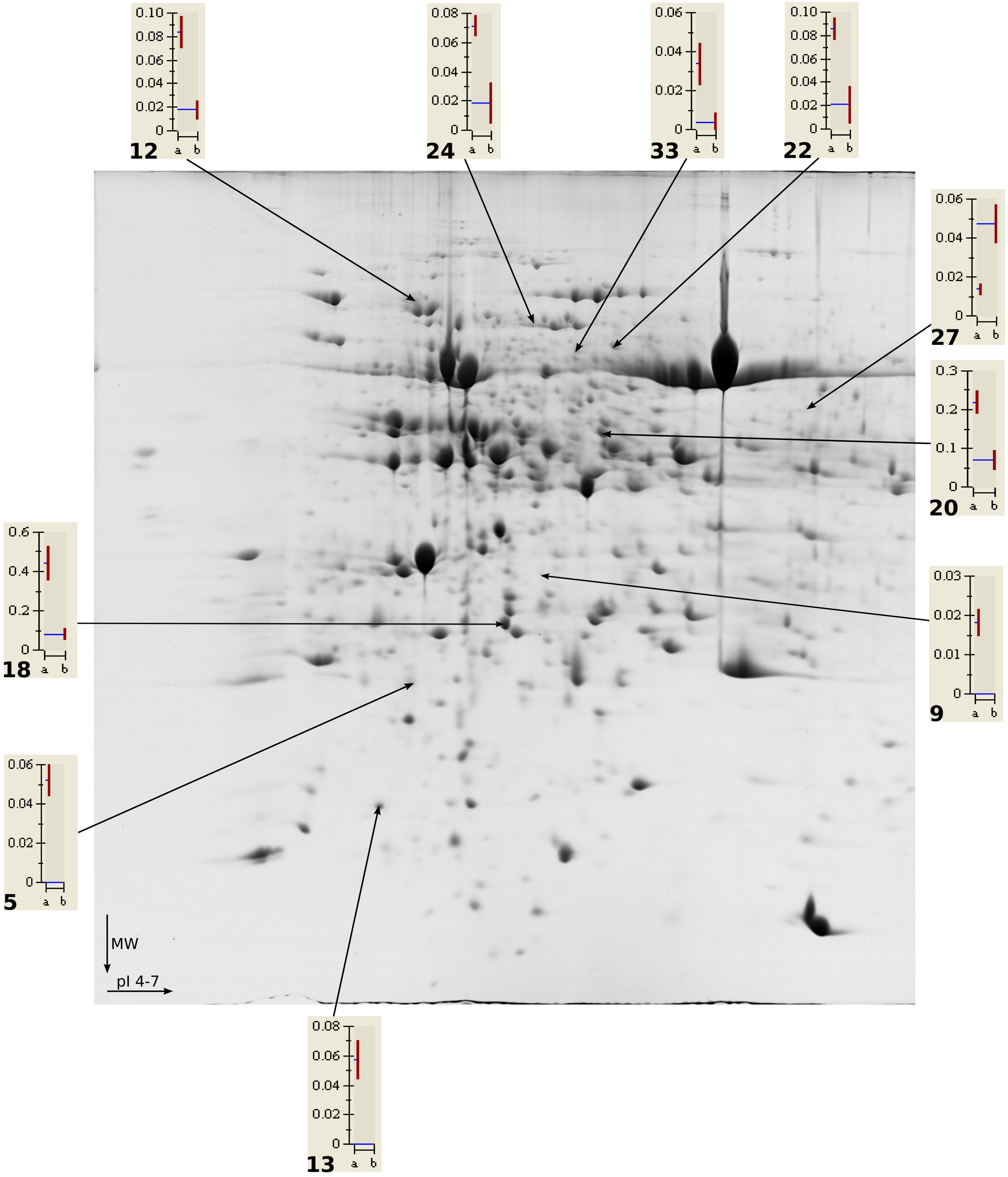

Supplementary Fig. 2. Representative 2-DE gel obtained for the **fine roots** of *S. viminalis* grown on a control soil or tannery waste. Differentially abundant protein spots (at least twofold variation at $p < 0.05$) identified with MS analysis are marked with arrows. Histograms show the distribution of relative spots volumes in each growth conditions (a = tannery waste, b = control soil).

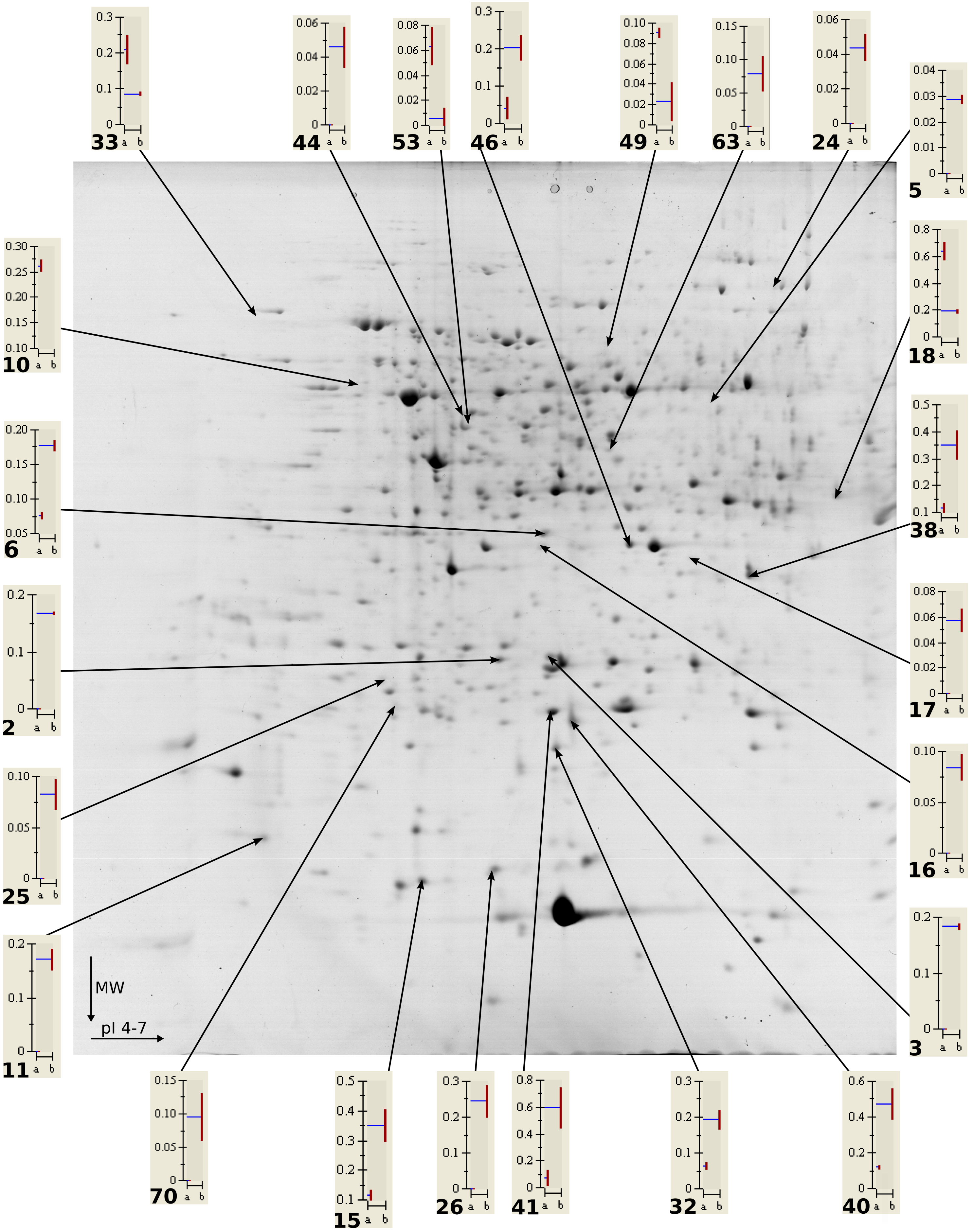

Supplement: Supplementary file 1 — (PDF 13095 kb) [file 11356_2016_7026_MOESM1_ESM.pdf]
